# ELF3 Is a Target That Promotes Therapeutic Efficiency in EGFR Tyrosine Kinase Inhibitor-Resistant Non-Small Cell Lung Cancer Cells via Inhibiting PKCί

**DOI:** 10.3390/ijms222212287

**Published:** 2021-11-13

**Authors:** Jeon-Soo Lee, Young Eun Choi, Sunshin Kim, Ji-Youn Han, Sung-Ho Goh

**Affiliations:** 1 Division of Cancer Biology, Research Institute, National Cancer Center, 323 Ilsan-ro, Goyang 10408, Korea; raphael@ncc.re.kr (J.-S.L.); 75162@ncc.re.kr (Y.E.C.); 2 Division of Rare and Refractory Cancer, Research Institute, National Cancer Center, 323 Ilsan-ro, Goyang 10408, Korea; ksunshin@ncc.re.kr (S.K.); jymama@ncc.re.kr (J.-Y.H.)

**Keywords:** ELF3, non-small cell lung cancer, EGFR, EGFR-TKI resistance, PKCί, auranofin

## Abstract

(1) Background: Mutations in epidermal growth factor receptor (EGFR) proteins account for many non-small cell lung cancers (NSCLCs), and EGFR tyrosine kinase inhibitors (TKIs) are being used as targeted therapeutics. However, resistance to TKIs continues to increase owing to additional mutations in more than half of the patients receiving EGFR TKI therapy. In addition to targeting new mutations with next-generation therapeutics, it is necessary to find an alternative target to overcome the challenges associated with resistance. (2) Methods: To identify potential alternative targets in patients with NSCLC undergoing targeted therapy, putative targets were identified by transcriptome profiling and validated for their biological and therapeutic effects in vitro and in vivo. (3) Results: ELF3 was found to be differentially expressed in NSCLC, and ELF3 knockdown significantly increased cell death in K-Ras mutant as well as in EGFR L858R/T790M mutation harboring lung cancer cells. We also found that auranofin, an inhibitor of protein kinase C iota (PKCί), a protein upstream of ELF3, effectively induced cell death. (4) Conclusions: Our study suggests that blocking ELF3 is an effective way to induce cell death in NSCLC with K-Ras and EGFR T790M/L858R mutations and thus advocates the use of auranofin as an effective alternative drug to overcome EGFR TKI resistance.

## 1. Introduction

Lung cancer was the second most commonly diagnosed cancer (11.4%) and the leading cause of cancer-related deaths (18.0%) worldwide in 2020 [1]. Non-small cell lung cancer (NSCLC) is a major subtype of lung cancer, and the main cause of NSCLC at the molecular level in Asian populations is an EGFR exon 19 in-frame deletion or L858R point mutation, which constitutively activate its receptor tyrosine kinase activity [2]. To target EGFR mutations, first-generation EGFR tyrosine kinase inhibitors (TKIs), such as gefitinib and erlotinib, have been developed and applied as targeted therapies. However, NSCLC patients treated with first-generation EGFR TKIs develop resistance in 10–19 months by acquisition of a second point mutation in the kinase domain of EGFR (T790M). The T790M mutation accounts for approximately 50–60% of acquired resistance and abolishes the effect of first-generation TKI by distorting the ATP-binding pocket to block the binding of the EGFR TKI. To overcome acquired mutation-based resistance, second-generation EGFR TKIs (e.g., afatinib) and third generation TKIs (e.g., osimertinib) have been developed. However, additional mutations (e.g., EGFR C797S) rendered the newly developed drugs ineffective [3]. Thus, it is necessary to develop new strategies to overcome the challenges associated with targeted drugs for EGFR mutants that are rendered ineffective due to the occurrence of unexpected mutations.

E74-like ETS transcription factor 3 (ELF3) was discovered as a novel epithelial ETS gene expressed in humans as well as in murine species [4] and acts as a transcriptional activator in many epithelial cancers [5,6]. ELF3 was reported to inhibit invasion of oral squamous cell carcinoma [7]; however, its overexpression was reported to be associated with the promotion of tumor growth in lung [8], poor cancer prognosis in colorectal cancer [9], copy number gain in breast cancer [10], and reported to facilitate tumor cell growth and metastasis in NSCLC [11]. In particular, ELF3 was also reported to be a potential biomarker for detecting circulating tumor cells in NSCLC [12] and has been suggested to be a prognostic biomarker in lung adenocarcinoma (LUAD) [13]. Despite strong evidence for its role in LUAD, there is no feasible therapeutic strategy to inhibit ELF3 expression or activity. Intriguingly, NOTCH is required for tumor initiation in LUAD, and ELF3 is recruited to the NOTCH3 promoter via phosphorylation by protein kinase C iota (PKCί) [14]. Auranofin (ANF), a drug approved for the treatment of rheumatoid arthritis (RA), was suggested to inhibit PKCί and has been tested in a K-Ras mutant lung cancer cell line [14]. ANF is an US Food and Drug Administration (FDA)-approved oral gold-containing compound that shows dose-dependent anti-cancer efficiency on a colon cancer cell line (DLD-1) [15]. In addition, a combination of 1 μM ANF with no cytotoxicity and celecoxib which inhibits COX-2 showed an apparent synergystic effect on various colorectal cancer cell lines [15]. However, its role in EGFR L858R/T790M mutant lung cancer cells has not yet been examined.

In this study, we analyzed the transcriptome profiles of patient-derived cells (PDCs) from NSCLC to identify patient-specific therapeutic targets and found elevated expression of ELF3 in some PDCs. To determine the effect of ELF3 inhibition in EGFR TKI resistance-related mutation, we tested the effects of ELF3 knockdown mediated by siRNA and treatment with ANF to inhibit the ELF3 upstream effector PKCί in H1975 cells in which EGFR harbors the L858R/T790M mutations found in first-generation EGFR TKI-resistant patients and compared it with the effects in K-Ras mutant A549 cells. In addition, we assessed the synergistic effect of ANF when it was applied in combination with the first-generation EGFR TKI, gefitinib, in vitro and in vivo. Here, we demonstrate that ELF3 overexpression is a potential marker for categorizing patients, and ANF represents an alternative therapeutic either alone or in combination to improve the treatment outcomes of first-generation EGFR TKI resistant patients.

## 2. Results

### 2.1. Transcriptome Analyses of PDCs from Lung Cancer Patients

Patients were categorized according to histology, driver mutations, and disease stage (Supplementary Table S1). Histologically, most of the patient samples were NSCLC (nine adenocarcinomas and one sarcomatoid), and two patients with small cell lung cancer with extensive diseases were also included. Driver mutations included EGFR mutations (L858R and exon 19 deletion) in six PDCs and ALK fusion in two PDCs. Except for one case of stable disease (SD), most PDCs were from patients with progressive disease (PD) or partial response (PR) to therapies. Among the PDCs, NCCLu-009 and 089 were baseline samples without treatment, and the remainder were treated and resistant. NCCLu-045 and 049 were derived from the pleural effusion of a patient with resistance to first-generation EGFR TKI (gefitinib) who received multiple chemotherapies; however, its growth pattern was different in suspended or adherent cultures, respectively. NCCLu-064 and 088 were longitudinal samples before and after the first therapy for small cell lung cancer with irinotecan-platinum-based first-line therapy. NCCLu-089 and 119 were baseline and post-ALK inhibitor (alectinib) therapy samples, respectively.

**Figure 1:**
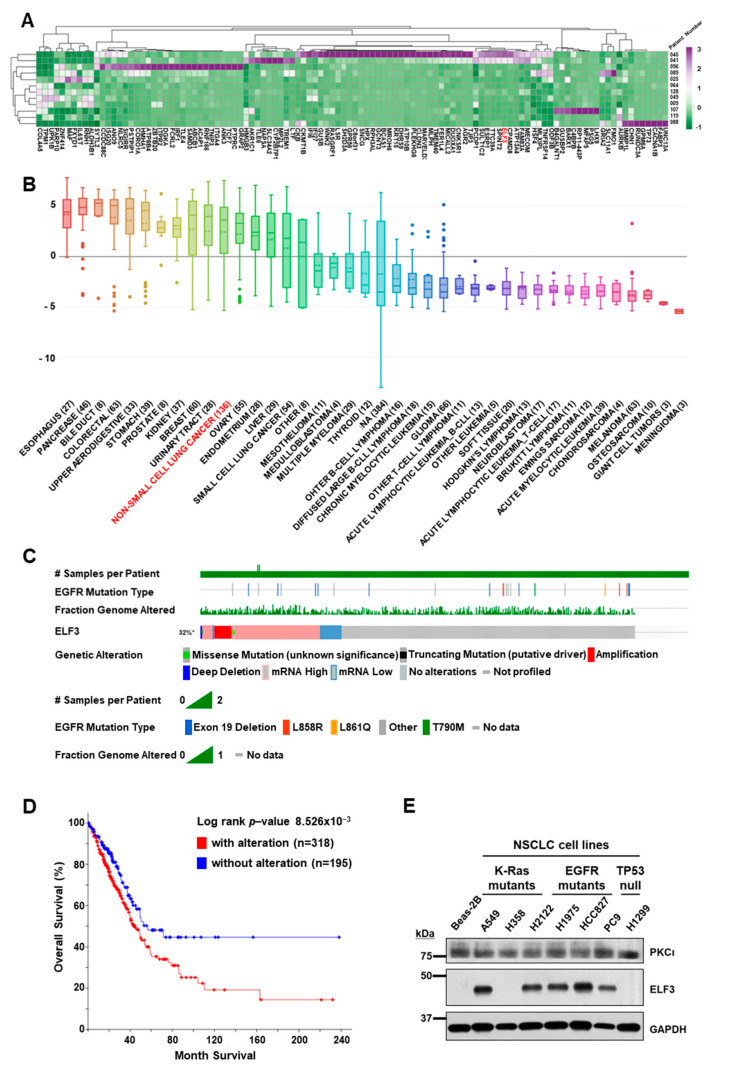
Transcriptome analyses of patient-derived cells from cases with lung cancer. (**A**) Transcriptomic analysis of 12 PDCs showing resistance to first-generation EGFR TKI therapies (expression difference > two-fold, *p* < 0.01). (**B**) Relative mRNA level of *ELF3* in NSCLC cell lines in the Cancer Cell Line Encyclopedia database (CCLE). (**C**) Alteration of ELF3 expression level in lung adenocarcinoma (LUAD) patients (*n* = 586, data were obtained from cBioPortal). (**D**) Overall survival rate of patients with alteration of ELF3 expression (*p* < 0.001, data were processed from cBioPortal). (**E**) Comparison of ELF3 level in normal bronchial epithelial cell line (Beas-2B) and several lung cancer cell lines. A total of 10 μg of protein was loaded into wells for each cell lysate. GAPDH served as a loading control.

Transcriptomes of the 12 PDCs were analyzed by RNAseq using the HiSeq2500 platform (Illumina, San Diego, CA, USA). In this study, we focused on the genes that increase resistance to first-generation EGFR TKI therapies. We selected a total of 112 differentially expressed genes that showed more than a two-fold difference (*p* < 0.01) in patients with EGFR (L858R) mutation and conferred resistance to EGFR TKI. The mRNA expression levels of these genes were clustered by unsupervised hierarchical clustering using ClustVis software [16], which revealed ten highly expressed genes in EGFR TKI-resistant patients with EGFR (L858R) mutant (NCCLu-041 and 045) (Figure 1A). Gene family analysis of these genes identified ELF3 as a transcription factor from the Molecular Signature Database (MSigDB) [17]. 

A high expression of *ELF3* mRNA was found in 136 NSCLCs cell lines in the Cancer Cell Line Encyclopedia (CCLE) database [18] similar to that observed in breast, urinary tract, or ovarian and endometrial cancers. However, small cell lung cancer showed lower expression of *ELF3* mRNA than NSCLC (Figure 1B). 

To assess the relevance of the ELF3 gene in lung cancer, we analyzed the aberrant status of ELF3 in the TCGA Firehose Legacy LUAD dataset (*n* = 586). Based on RNAseq data from patients with LUAD, the ELF3 gene was found to be altered in 32% of the cases, and most of the alterations involved a high expression of the mRNA and amplification of the gene itself (Figure 1C). The frequency of amplification was relatively lower in the TCGA LUAD dataset than in previous reports, whereas an elevated expression of ELF3 mRNA was observed in 26% of the cases. Both the alteration and amplification of ELF3 expression appeared to be independent of the EGFR mutation status. The overall survival of patients with alteration of ELF3 expression was significantly lower than that of patients without alteration (*p* < 0.001) (Figure 1D). 

To explore the function of ELF3 in lung cancer, we examined its protein expression in several lung cancer cell lines (Supplementary Table S2) and found that ELF3 was detectable in most of the cell lines regardless of the mutational status of EGFR or K-RAS (Figure 1E). However, it was not detected in some lung cancer cells, including H358 and H1299, as well as in Beas-2B, a normal lung cell. Therefore, we selected A549 and H1975 cell lines for further analyses of ELF3 function.

### 2.2. ELF3 Knockdown Induced Cell Death and Reduced Cell Cycle Progression in Lung Cancer Cells

**Figure 2:**
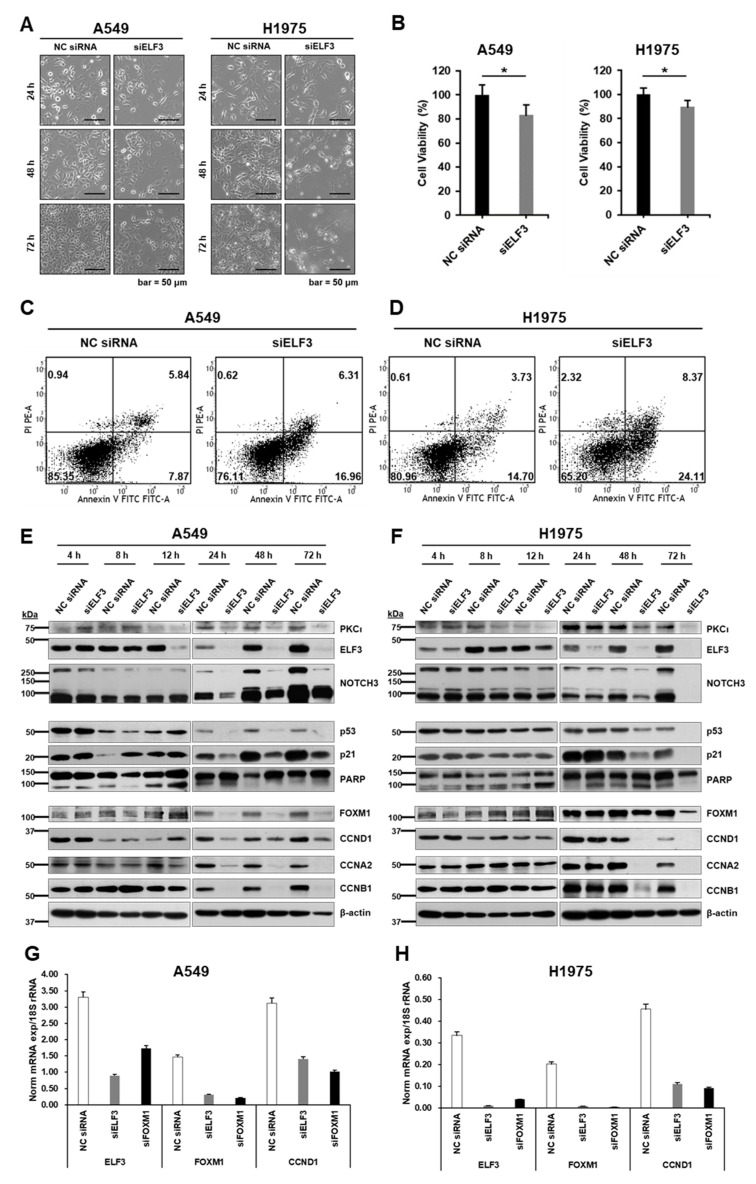
Effects of ELF3 knockdown in lung cancer cells. (**A**) Microscopic images of A549 (**left** column) and H1975 (**right** column) cells transfected with NC siRNA and siELF3. (**B**) Cell viability of A549 (**left**) and H1975 (**right**) cells measured by WST-1 assay (statistical significance of NC siRNA versus siELF3 * *p* < 0.05). Annexin-V assay performed with A549 (**C**) and H1975 (**D**) cells 72 h after siRNA transfection. Immunoblotting of siRNA-transfected A549 (**E**) and H1975 (**F**) cells for levels of PKCί (75 kDa), ELF3 (44 kDa), NOTCH3 (full-length 270 kDa, cleaved intracellular domain 90 kDa), p53, p21, PARP (full-length 116 kDa, cleaved 89 kDa), several cyclins, and β-actin. qRT-PCR analysis results showing mRNA expression of ELF3, FOXM1, and CCND1 in A549 (**G**), and H1975 cells (**H**) treated with siELF3 for 48 h. 18S rRNA was used as an endogenous control.

ELF3 was reported to be activated following phosphorylation by protein kinase C iota (PKCί), and it then transmits the signal to NOTCH3, which is associated with cell survival and chemoresistance [11,14]. Therefore, to assess the effect of ELF3 on lung cancer cell survival, we knocked down the expression of ELF3 by transfecting ELF3 siRNA into A549 (with K-Ras mutation) and H1975 (with EGFR L858R/T790M double mutation) lung cancer cells for 72 h. Compared to the negative control (NC) siRNA treatment, cells with siELF3-mediated ELF3 knockdown showed reduced cell confluence in both cell lines (Figure 2A). Based on this observation, we analyzed cell viability to assess the role of ELF3 in cell proliferation. Cell viability was measured using the WST-1 assay and showed significantly reduced viability in siELF3-treated cell lines (*p* < 0.05) (Figure 2B). Next, we examined cell death induced by ELF3 knockdown using Annexin-V assay. In ELF3-knockdown A549 cells (Figure 2C), the proportion of pre-apoptotic cells increased more than two-fold compared to the NC siRNA control transfected cells. Similarly, in ELF3 knockdown H1975 cells, both apoptotic and pre-apoptotic cells were increased almost two-fold compared to that in the NC siRNA control transfected cells (Figure 2D). In this context, the levels of proteins involved in cell death and cell cycle were analyzed. Early activation of NOTCH3, p53, p21, and PARP was found to fade away as ELF3 knockdown persisted. In the protein cascade involved in cell death, an increased level of p53 is known to be a key factor that triggers apoptosis. In A549 cells, the levels of p53 and p21 sharply increased at 12 h of incubation to induce cell death. After this point, cleaved PARP (89 kDa) gradually increased, up to 72 h of incubation, at which point it appeared to disappear because most cells were dead. 

The cell cycle-related proteins cyclin A2, B1, and D1 were reduced in a similar fashion. FOXM1, an oncogenic transcription factor that activates cell proliferation-related genes (Figure 2E), was also similarly reduced. In H1975 cells, the levels of p53 and p21 were slightly increased at 24 h of incubation. Along with the change in p53 level, cleaved PARP (89 kDa) also increased. Similar to the 72 h incubation point in A549 cells, cleaved PARP was decreased at the same time point in H1975 cells. Cyclin A2, B1, and D1 as well as PKCί were decreased at a later time point compared to the cell death-related proteins. The decrease in cyclins and PKCί paralleled the level of FOXM1 in both cell lines (Figure 2F). To confirm whether ELF3 also regulates transcriptional activation of FOXM1, we examined the change in mRNA levels of *FOXM1* and *CCND1* by quantitative reverse transcription-polymerase chain reaction (qRT-PCR) and found that FOXM1 and CCND1 mRNA levels were also decreased following ELF3 knockdown in both A549 (Figure 2G) and H1975 cells (Figure 2H) (Supplementary Table S3). These data suggest that ELF3 knockdown affected cell proliferation by reducing the expression of cyclin proteins and increased cell death in both K-Ras mutant A549 and EGFR L858/T790M mutant H1975 cells.

### 2.3. ANF Represses ELF3 via Inhibition of PKCί 

**Figure 3:**
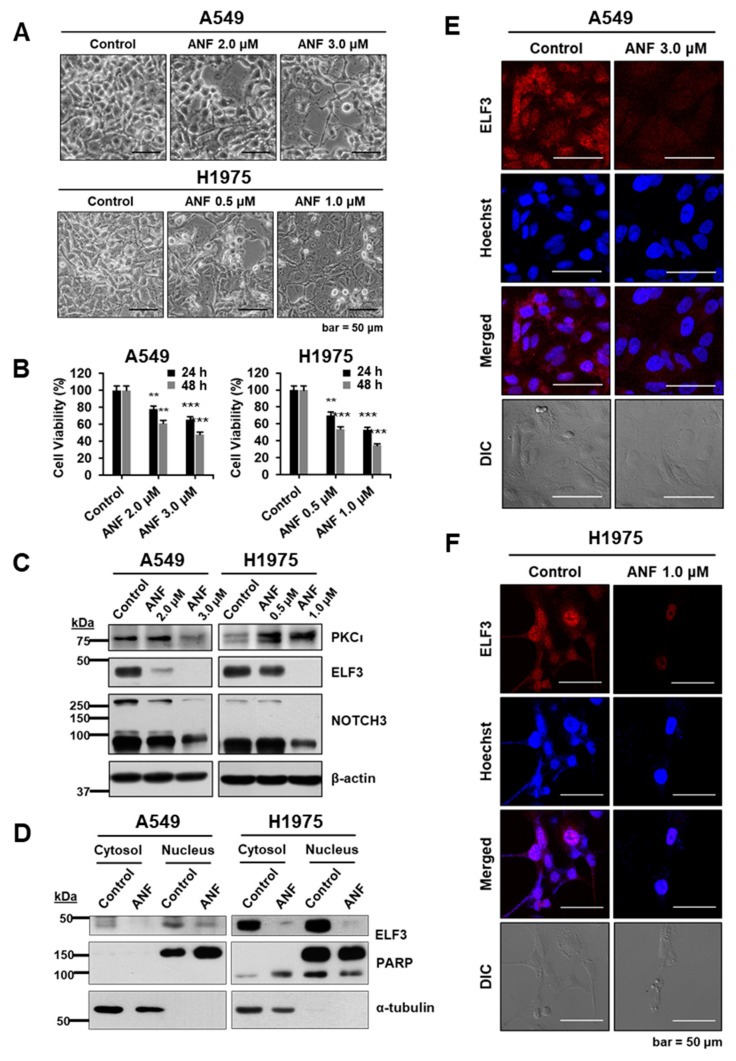
ANF represses ELF3 via inhibition of PKCί. (**A**) Microscopic images of A549 (**upper** panel) and H1975 (**lower** panel) cells treated with ANF for 48 h. (**B**) Cell viability of auranofin (ANF)-treated A549 (**left**) and H1975 (**right**) cells measured by WST-1 assay (** *p* < 0.01; *** *p* < 0.001, cells were treated with ANF for 48 h). (**C**) ANF-treated A549 and H1975 cells were immunoblotted for PKCί (75 kDa), ELF3 (44 kDa), NOTCH3 (full-length 270 kDa, cleaved intracellular domain 90 kDa) and β-actin (cells were treated with ANF for 48 h). (**D**) Immunoblotting of nuclear fraction of ANF-treated A549 and H1975 cells for ELF3 (44 kDa), PARP (nuclear fraction) and α-tubulin (cytosolic fraction) (cells were treated with ANF for 48 h). Confocal microscopic images of immunocytochemistry (ICC) staining of ANF-treated A549 (**E**) and H1975 (**F**) (Red; 594 nm and Hoechst; 350 nm).

ANF is an inhibitor of PKCί [14]. It is also used as a therapeutic agent for RA. However, given that the expression of ELF3 is upregulated in several K-Ras mutant LUADs and lung squamous cell carcinomas (LSCCs), whether auranofin has therapeutic potential to repress ELF3 through inhibition of PKCί activity in EGFR mutant LUAD remains unclear [19]. We treated A549 and H1975 cells with ANF for 48 h. Similar to siELF3-treatment, ANF induced a reduction in cell confluence in both A549 and H1975 cells (Figure 3A). To investigate its repressive effect on cell proliferation, we measured cell viability and found that it was decreased in an ANF concentration-dependent manner in both the cell lines (Figure 3B). In EGFR L858/T790M mutant H1975 cells, cell viability was markedly downregulated at a lower concentration of ANF (0.5 or 1.0 μM) than A549 cells (2.0 or 3.0 μM). 

We examined the changes in protein levels of PKCί, ELF3, and NOTCH3, which is a target gene of ELF3. ANF effectively reduced the level of ELF3 and NOTCH3. The level of PKCί was decreased in A549 cells in an ANF concentration-dependent manner, but it was increased in H1975 cells (Figure 3C). Although ANF is an inhibitor of the kinase activity of PKCί, its inhibition appears to be independent of changes in the PKCί protein level. 

PKCί functions as a protein kinase to phosphorylate ELF3 during cell proliferation. Phosphorylated ELF3 is translocated into the nucleus and binds to the promoter region of its target genes, including NOTCH3, to promote transcription. However, in this study, when PKCί was inhibited by ANF, the nuclear translocation of ELF3 was reduced in both A549 and H1975 cells (Figure 3D). Based on the immunoblot of nuclear fractions, the delocalization of ELF3 from the nucleus following ANF treatment was clearly demonstrated in both cell lines by the immunocytochemistry (ICC) stained images (Figure 3E). These data indicate that inhibition of PKCί by ANF induces the inactivation and delocalization of ELF3 and eventually affects the rate of cell proliferation.

### 2.4. ANF and GEF Synergistically Inhibit TKI Resistance in Cells 

Various TKIs have been developed to target specific mutations for the treatment of lung cancers, the main molecular cause of which is mutation in the tyrosine kinase domain of EGFR. However, patients who are resistant to first-generation TKIs such as gefitinib (GEF) have double EGFR mutations T790M/L858R in EGFR, which confers a therapeutic refractory period. Although 2nd and 3rd generation EGFR TKIs are available, unexpected mutations can emerge to induce resistance and render them useless. Thus, there is an urgent need to overcome such resistance, regardless of the EGFR mutations in patients. As we demonstrated in the previous experiment, inhibition of PKCί by ANF treatment resulted in a delocalization and reduction of ELF3 in both A549 and H1975 cells. This regulation of ELF3 via PKCί inhibition is independent of the EGFR/Ras/MAPK pathway, which is a key pathway that promotes oncogenesis in NSCLC [13]. Therefore, ANF-mediated inhibition of PKCί represents a novel therapeutic strategy for NSCLC patients with high ELF3 levels regardless of mutations in EGFR or other main oncogenes such as K-Ras. To evaluate the effectiveness of ANF and GEF as single agents or in combination, we measured the rates of cell proliferation using the WST-1 assay. 

**Figure 4:**
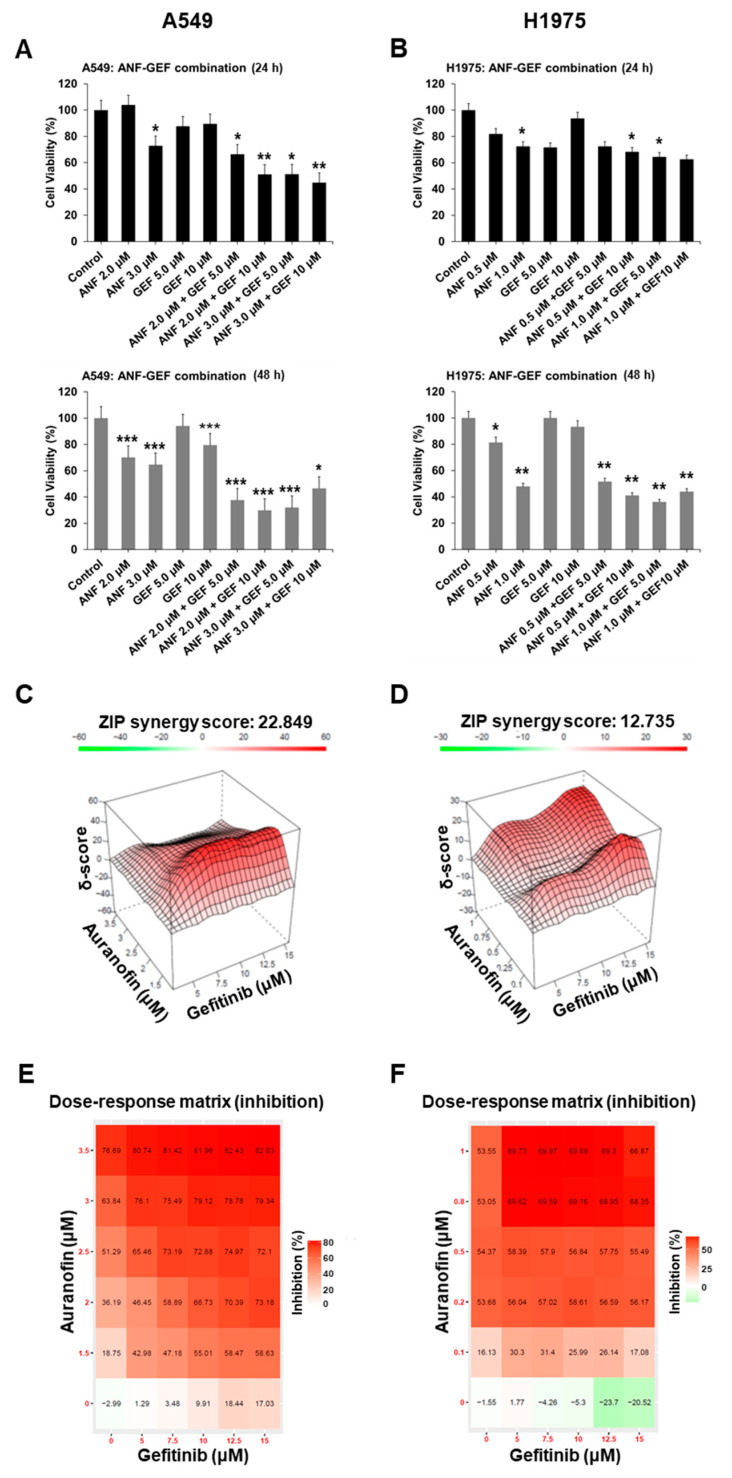
Auranofin synergistically inhibits TKI resistance in cells. (**A**) Cell viability of single agent-treated and ANF-GEF combination-treated A549 (**A**) and H1975 (**B**) cells measured by WST-1 assay (statistical significance of control versus combination-treated *; *p* < 0.05. **; *p* < 0.01, ***; *p* < 0.001, cells were treated with ANF for 48 h). Synergistic delta (δ) score of ANF-GEF combination treatment was 22.849 in A549 (**C**) (synergistic; δ > 10) and 12.735 for H1975 cells (**D**). Inhibition (%) of cell growth in A549 (**E**) and H1975 cells (**F**) from Synergy Finder 2.0 [18].

Treatment of A549 cells with 5.0 μM GEF was ineffective. GEF became effective when the cells were incubated for 48 h at a 10.0 μM concentration (*p* < 0.001) (Figure 4A). However, in H1975 cells, GEF showed no significant effect even at higher concentrations compared to A549 cell (5.0 or 10 μM) (Figure 4B). ANF treatment of A549 cells caused a significant reduction in cell proliferation at 24 h (3.0 μM; *p* < 0.05) and 48 h (2.0 μM; *p* < 0.001) (Figure 4A). However, its effectiveness in H1975 cells was much lower than that in A549 cells. Treatment with 0.5 μM ANF reduced cell viability to 80% of NC after 24 h and to lower than 50% after 48 h with 1.0 μM treatment (*p* < 0.01) (Figure 4B). These results suggest that ANF treatment is effective in reducing cell viability even in NSCLC cells that harbor K-Ras and concurrent EGFR mutations, and it is even more effective compared to GEF. 

To assess the effect of combinatorial treatment, we treated cells with a combination of the two drugs. In A549 cells, the combination of ANF with GEF was more effective in reducing cell viability than single drug treatment (Figure 4A). Similarly, in H1975 cells, the effect of combinatorial treatment was more significant at 48 h (*p* < 0.01) than at 24 h (*p* < 0.05) compared to the NC (Figure 4B). To determine whether the combination treatment was synergistic or additive, we analyzed the synergistic effect on inhibition of cell viability using Synergy Finder 2.0 [20], following treatment of A549 and H1975 cells with ANF and GEF. In A549 cells, a synergistic effect was found at a lower concentration of ANF (1.5 μM) than when it was combined with GEF in a wide range of concentrations (Figure 4C). In contrast, in H1975 cells, a synergistic effect was found at both low (0.25 μM) and high (1.0 μM) ANF concentration when combined with high GEF concentration (12.5 μM) (Figure 4D). A Synergy Finder ZIP score > 10 is considered to be synergistic, and in our study, we found that the ZIP scores were 22.849 and 12.735 for A549 and H1975 cells, respectively (Figure 4C,D). Thus, these data suggest that the combination of ANF and GEF is more effective and has a synergistic effect compared to single drug treatment.

In contrast to their synergistic effectiveness, inhibition of cell proliferation was observed at high concentrations of the two reagents (Figure 4E,F). In A549 cells, cell proliferation was inhibited by up to 82.83% when 3.5 μM ANF and 15.0 μM GEF were combined (Figure 4E). Similarly, in H1975 cells, cell proliferation was inhibited up to 69.89% when 1.0 μM of ANF and 10.0 μM of GEF were used in combined treatment (Figure 4F). In particular, although H1975 cells were resistant to GEF, the combination of ANF and GEF appeared to synergistically inhibit the proliferation of H1975 cells. These data demonstrate that the combination of ANF and GEF have increased therapeutic efficiency to overcome resistance via inhibition of the EGFR-independent pathway.

### 2.5. ANF and GEF Combination Induces Apoptosis and Repression of Cell Cycle 

**Figure 5:**
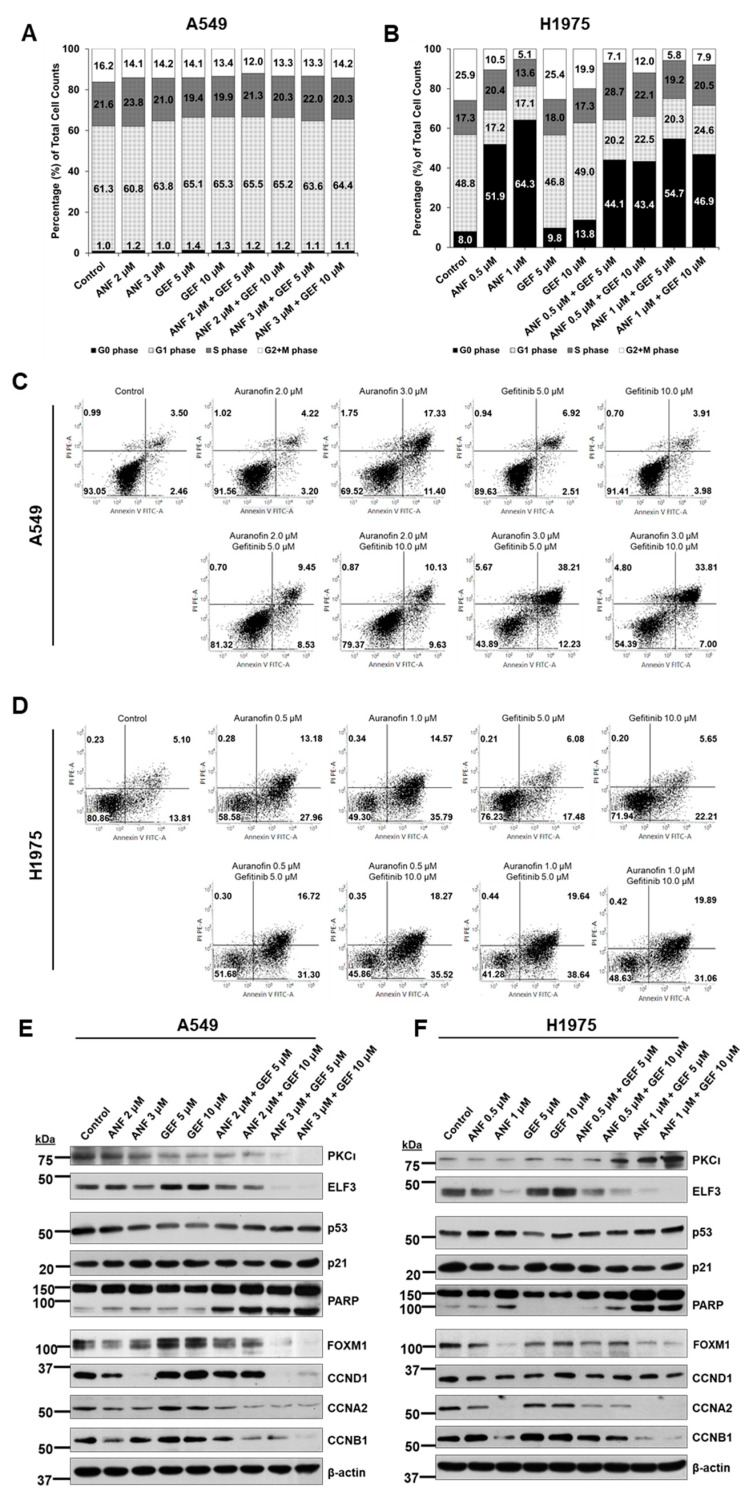
ANF and GEF induce apoptosis and repression of cell cycle. Cell cycle analysis in A549 (**A**) and H1975 (**B**) cells. Annexin V analysis of apoptosis in A549 (**C**) and H1975 (**D**) cells. Immunoblotting of ELF3 (44 kDa), NOTCH3 (full-length 270 kDa, cleaved intracellular domain 90 kDa), FOXM1 (100 kDa), c-Myc (52 kDa), and cyclins in A549 (**E**), and H1975 (**F**) cells treated with ANF, GEF, or ANF/GEF.

Given our results that the ANF-GEF combination synergistically inhibits cell proliferation, we analyzed the changes in cell cycle and apoptosis at various concentrations of ANF and GEF in A549 and H1975 cells. Cell cycle analysis of A549 cells revealed that regardless of the concentration of ANF and GEF alone, or a combination, the G0 phase of the cell cycle did not change significantly. The S and G2/M phases were slightly decreased compared to the control (61.3%) (Figure 5A). In contrast, similar analysis of H1975 cells revealed that the G0 phase was markedly increased depending on ANF concentration (51.9% for 0.5 μM and 64.3% for 1.0 μM) compared to the DMSO control (8.0%). However, GEF treatment did not cause any significant increase (9.8% at 5 μM and 13.8% at 10 μM). The ANF/GEF combination also increased the G0 phase from 43.4% to 54.7%, depending on the concentration used (Figure 5B). These results suggest that ANF-mediated inhibition of cell cycle regulation in EGFR double mutant H1975 cells was more effective than that in K-Ras mutant A549 cells. 

Then, we analyzed apoptosis in cells treated with the same dose of drugs as that used in the cell cycle analysis. The frequency of apoptotic population in A549 cells treated with 5.0 μM (6.91 + 2.51%) or 10.0 μM (3.91 + 3.98%) GEF was not significantly increased compared to the control (3.50 + 2.46%). Although treatment with 2.0 μM ANF was ineffective (4.22 + 3.20%), treatment with 3.0 μM ANF was significantly effective (17.33 + 11.40%) (Figure 5C). In particular, when A549 cells were treated with a combination of ANF and GEF, the proportion of apoptotic cells were markedly increased by approximately seven-fold (38.21% + 12.23%, ANF 3.0 μM and GEF 5.0 μM) compared to that in the control (3.50% + 2.46%). Although A549 has a K-Ras mutation but no EGFR mutation, it is known that A549 is resistant to GEF with an IC_50_ of >10 μM [21]. Our apoptotic analysis data from the A549 cells indicate that ANF-GEF combination may be applied effectively in patients with resistance against first-generation EGFR TKIs, such as GEF, without a mutation in EGFR. In H1975 cells, the increment in the proportion of apoptotic cells was not that markedly high even at higher doses of GEF (10.0 μM; 5.65 + 22.21%) compared to the control (5.10% + 13.81%). However, both 0.5 μM (13.18 + 27.96%) or 1.0 μM (14.57 + 35.79%) ANF showed dramatic effect in inducing cell death compared to GEF single treatment. The combination treatment with ANF and GEF resulted in apoptosis of > 48% at all concentrations. Specifically, the combination of ANF (1.0 μM) and GEF (5.0 μM) showed the highest increase in apoptosis (19.64 + 38.64%). These data suggest that while ANF single agent treatment is effective in inducing cell death, an ANF/GEF combination synergistically induces cell death in concurrent p53 and EGFR T790M/L858R mutant H1975 cells. This result was consistent with the increased apoptosis mediated by ELF3 knockdown in A549 and H1975 cells (Figure 2C,D).

Immunoblot analysis of the changes in the levels of downstream cell cycle-related proteins following drug treatment revealed that ANF/GEF combination decreased the levels of several target proteins in A549 and H1975 cells (Figure 5E,F). We found that ELF3 knockdown reduced the expression of CCNA2, CCNB1, and CCND1 (Figure 2E,F). Similarly, treatment with ANF/GEF combination decreased the levels of CCNA2, CCNB1, and CCND1 compared to the NC and single agent-treated groups in both cell lines. We also examined the changes in FOXM1 level because FOXM1 is known to regulate cyclin protein levels [22]. As expected, similar to the results of ELF3 knockdown, FOXM1 was decreased in both ANF treatment and ANF/GEF combination treatment. In A549 and H1975 cells, the level of FOXM1 was markedly reduced in the ANF/GEF combination treatment (Figure 5E,F). In A549 and H1975 cells, cleaved PARP levels were also increased in the ANF/GEF combination. Although ANF treatment (3.0 μM in A549 cells, 1.0 μM in H1975 cells) slightly increased cleaved PARP levels, the combination treatment induced the levels of cleaved PARP further. ANF treatment has been reported to increase the level of cleaved Caspase-3, which cleaves PARP, in murine osteosarcoma cells [23]. As cleaved PARP is a marker of apoptosis, these results suggest that the combination of ANF/GEF induces apoptosis of cells, and these results are consistent with the apoptotic analysis data (Figure 5C,D).

### 2.6. ANF and GEF Combination Reduces Cell Growth in an In Vivo Mouse Model

**Figure 6:**
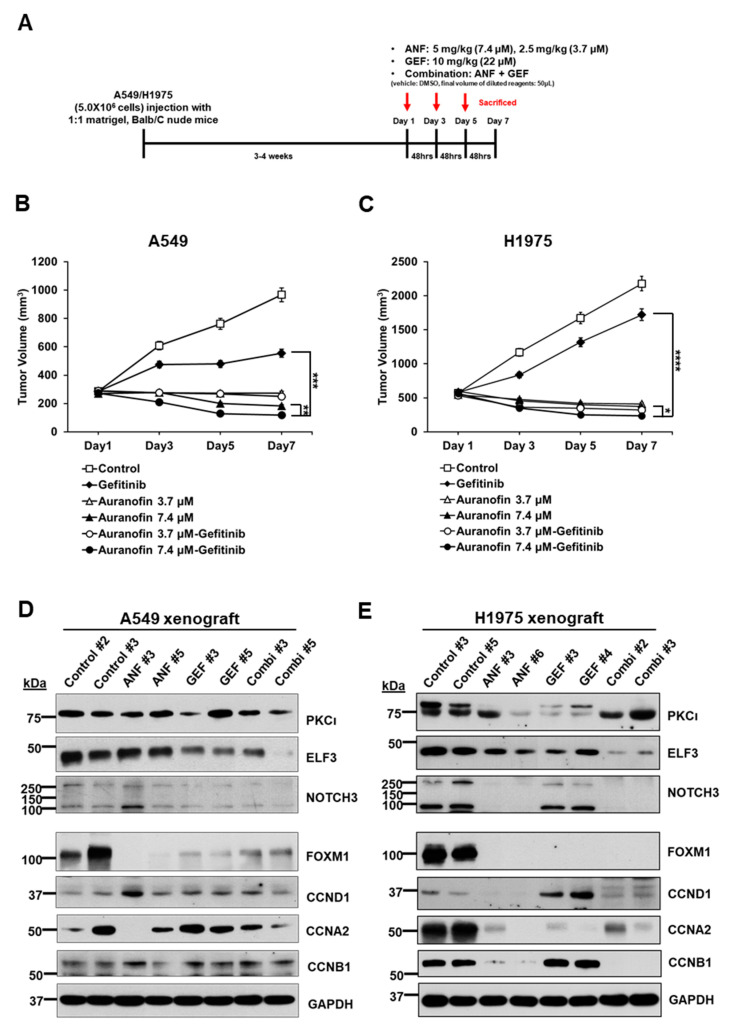
ANF and GEF reduce cell growth in an in vivo mouse model. (**A**) Schematic diagram showing the protocol used in the xenograft mouse model. A total of 5.0 × 10^6^ cells were injected in 1:1 ratio with Matrigel. A tumor size of A549 (**B**) (GEF versus ANF (7.4 μM)/GEF (22 μM) *** *p* < 0.001, ANF (7.4 μM) versus ANF (7.4 μM)/GEF (22 μM) ** *p* < 0.01), and H1975 (**C**) (GEF versus ANF (7.4 μM)/GEF (22 μM) **** *p* < 0.0001, ANF (7.4 μM) versus ANF (7.4 μM)/GEF (22 μM) * *p* < 0.05) xenografts. Immunoblotting of ELF3 (44 kDa), NOTCH3 (full-length 270 kDa, cleaved intracellular domain 90 kDa), FOXM1 (100 kDa), c-Myc (52 kDa), and cyclins in A549 (**D**) and H1975 (**E**) xenografts.

To evaluate the efficacy of the combination of the two drugs in vivo, we used a xenograft mouse model using BALB/c nude mice. After transplanting A549 or H1975 cells mixed with Matrigel (1:1 ratio) in mice, we waited until the tumor masses grew to approximately 200 mm^3^ and then injected the drugs either as single agents or in combination three times (on days 1, 3, and 5) intraperitoneally (Figure 6A). During the drug treatment period, we measured the size of tumor masses and recorded the body weight of mice to analyze the toxic physiological effects of the drug combination. The injection of ANF, GEF, or ANF/GEF combination did not affect the body weight of the xenograft mice harboring A549 (Supplementary Figure S1A) or H1975 (Supplementary Figure S1B) tumors. This result suggests that the drug combination had no toxic effect on the physiological status of mice. 

In A549 xenograft mice, the combination of ANF (7.4 μM)/GEF (22 μM) was more effective in causing a significant regression of tumor growth than single treatment of GEF (*p* < 0.001) or ANF (*p* < 0.01) (Figure 6B). Although another combination of ANF (3.7 μM)/GEF (22 μM) reduced 81.3% of tumor mass than single treatment of GEF, it was less effective than the combination of ANF (7.4 μM)/GEF (22 μM). The combination of ANF (7.4 μM)/GEF (22 μM) caused a significant regression of tumor growth than single treatment of GEF (*p* < 0.0001) or ANF (*p* < 0.01) (Figure 6C). In the H1975 xenograft mouse model, the H1975 tumor was highly susceptible to all ANF single treatments and combinations because its IC_50_ against ANF was in the range of 0.5–1.0 μM.

Next, we performed immunoblotting to detect the levels of the target proteins including PKCί, ELF3, NOTCH3, and cyclins. ELF3 was mainly decreased in the combination groups in A549 (Figure 6D) and H1975 (Figure 6E) xenografts. These results are similar to the data from the in vitro experiments. As observed in ELF3 knockdown or ANF treatment in vitro experiments in A549 and H1975 cells, cyclin A2, cyclin B1, and cyclin D1 were reduced in xenograft tumor tissues treated with ANF/GEF combination (Figure 6D,E). Since FOXM1 is a known regulator of the cell cycle and a downstream molecule of the EGFR/Ras/MAPK signaling pathway [20], we also analyzed the changes in FOXM1 in the xenograft tumor tissues. Immunoblotting data indicated that ANF affected FOXM1, and its regulation was independent of the mutation status of the EGFR/Ras/MAPK signaling pathway, which is more sensitive to TKIs such as GEF. In addition, as ELF3 knockdown appeared to reduce the mRNA levels of FOXM1 in A549 (Figure 2G) and H1975 cells (Figure 2H), ANF or ANF/GEF combination also resulted in a downregulation of FOXM1 in xenograft tumors.

## 3. Discussion

We examined the expression level of ELF3 mRNA in PDCs of patients with LUAD. ELF3 is an important factor associated with cell survival of lung cancer cells, and ANF is a potent inhibitor that represses cell growth and induces apoptosis of lung cancer cells through downregulation of the PKCί–ELF3 axis in K-Ras mutant A549 lung cancer cells [14]. In this study, we showed that the knockdown of ELF3 decreased cell proliferation and induced cell death in H1975 cells with concurrent EGFR mutations as well as in A549 cells. Furthermore, a combination of ANF and a TKI such as GEF synergistically inhibited the growth of lung cancer cells harboring specific mutations in EGFR that confer chemoresistance to TKIs. 

PKCί is a known moderator of chronic inflammation, such as RA and other inflammatory diseases. ANF is used to alleviate RA via inhibition of PKCί; however, its inhibitory mechanism has not been fully defined. The overexpression of PKCί was firstly reported in K-RAS-mutated LUAD, and its overexpression showed poor prognosis [24]. In a conditional PKCί knockout mouse model (*LSL-K-ras;Pkrcil^f/lf^*), which did not express PKCί in a lung-specific manner, oncogenesis of LUAD was markedly reduced [25]. Thus, PKCί has been considered to be an oncogenic moderator in LUAD. 

PKCί phosphorylates ELF3, and phosphorylated ELF3 is translocated into the nucleus to promote the transcription of its target genes. PKCί knockdown led to reduced levels of ELF3 and stem-like phenotypes in lung cancer cells, including A549 and H358 cells, and decreased ELF3 binding to the promoter region of NOTCH3 [13]. ELF3 is an ETS transcription factor. Various ETS transcription factors are related to tumor initiation [26]. ETV1, ETV4, and ETV5, which belong to the PEA3 group of genes [27], are types of ETS transcription factors that are known to promote several types of cancer such as Ewing-like sarcoma, gastric cancer, colorectal cancer, hepatocellular carcinoma and prostate cancer [28,29,30,31,32]. Most ETS transcription factors appear to play oncogenic roles in human cancers. ELF3 has also been reported to be an oncogenic ETS transcription factor that is amplified in LUAD but not in lung squamous carcinoma [13]. Although previous reports have suggested that the PKCί–ELF3 axis functions in the initiation of LUAD, there are no known therapeutics that target the pathways or oncogenic proteins in this axis. Therefore, ELF3 represents a promising therapeutic target to alleviate the resistance caused by EGFR mutations. 

In our study, we found that ANF effectively represses the PKCί–ELF3 axis and inhibits tumor growth of lung cancer cells harboring EGFR mutation relevant to 1st-generation EGFR TKI resistance. Consistent with previous reports [13,14], ANF reduced the level of ELF3 in K-Ras-related A549 cells by inhibiting PKCί. Interestingly, ANF treatment also decreased ELF3 levels in concurrent TP53 and EGFR double-mutant H1975 cells. These mutations are clinically important to overcome failure of targeted therapy because they are known to confer resistance to TKIs such as GEF. The combination of ANF and GEF inhibited cell growth and increased apoptosis in H1975 cells more efficiently than A549 cells. This finding indicates that ANF represents as an alternative therapeutic agent to increase the therapeutic efficiency in LUAD patients experiencing failure in targeted therapy owing to specific mutations in several key oncogenes.

Treatment with the ANF/GEF combination decreased the levels of cyclins, including cyclin A2, cyclin B1, and cyclin D1. Previous studies have reported that ANF reduces cancer cell stemness via inhibition of the PKCί–ELF3–NOTCH3 axis [14] and downregulates signaling pathways including the PI3K/Akt and ERK pathways [11,13]. However, there were not enough data to support that ANF directly regulates the cell cycle. We found that FOXM1 levels were reduced in A549 and H1975-xenografted mouse models as well as in in vitro assays (Figure 5E,F and Figure 6C). Since FOXM1 is a potent regulator of cyclins and is regulated by the EGFR/Ras/MAPK pathway, it is possible that either ANF alone or in combination with GEF decreases FOXM1 and its downstream molecules, including the cyclins. As mentioned in the results section, we also found that ELF3 knockdown decreased the level of FOXM1 (Figure 2E,F). RT-PCR analysis revealed that ELF3 knockdown also reduced the mRNA level of FOXM1 in A549 and H1975 cells (Figure 2G,H). As a transcription factor, ELF3 would be expected to transcriptionally regulate the expression of FOXM1; however, further studies are needed to reveal the exact mechanism.

**Figure 7:**
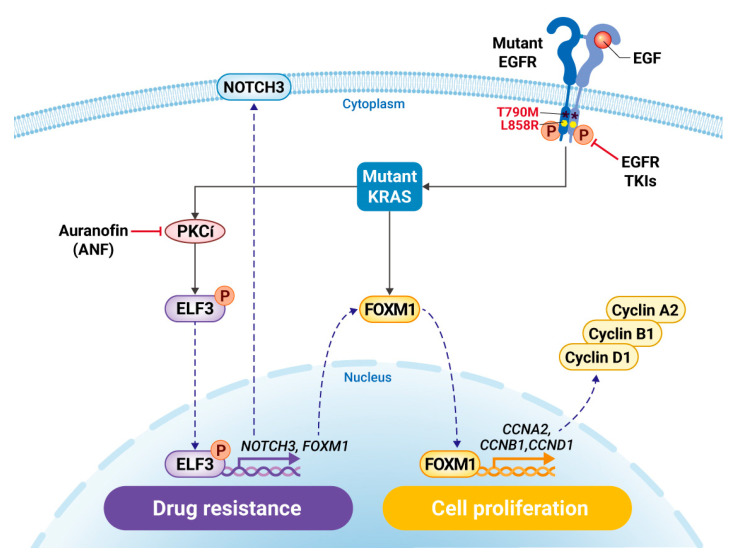
Proposed mechanistic model of auranofin and gefitinib combination.

In conclusion, it is proposed that inhibiting PKCί via ANF leads to the repression of ELF3 and its translocation into the nucleus. Reduced nuclear ELF3 fails to induce its target oncogenes, including NOTCH3 and co-regulating partners such as FOXM1 and c-Myc, independent of oncogenic signaling pathways (Figure 7). Thus, ANF represents a therapeutic strategy to increase the sensitivity of cells and overcome TKI resistance.

## 4. Materials and Methods

### 4.1. Transcriptome Analyses from Patient-Derived Cells

Twelve patient-derived cells (PDC) were established from pleural effusion of 10 lung cancer patients (Supplementary Table S1) and maintained for two passages in RPMI1640 medium supplemented with 10% fatal bovine serum (FBS) in a 37 °C incubator with 5% CO_2_. The transcriptome was analyzed by using total RNA purified from PDC and subjected to be analyzed on a HiSeq platform (Illumina, San Diego, CA, USA). Gene-set enrichment and gene family analyses were performed on a Molecular Signature Database (MSigDB) of the Broad Institute [17]. Unsupervised hierarchical clustering was per-formed using ClustVis [16]. This study was approved by an Institutional Review Board of National Cancer Center (Goyang, Korea; protocol number NCC 2016-0208).

### 4.2. Cell Lines and Maintenance

Beas-2B, A549, H358, H2122, H1975, HCC827, PC9, and H1299 were obtained from the American Type Culture Collection (Manassas, VA, USA) or Korean Cell Line Bank (Seoul, Korea). Beas-2B was maintained in Bronchial Epithelial Cell Growth Basal Medium (BEBM) (Cat. No. CC-3170, Clonetics^®^, Lonza Inc., Walkersville, MD, USA) with Bronchial Epithelial SingleQuots kit (Cat. No. CC-4175, Lonza Inc., Walkersville, MD, USA) as supplementary additives at 37 °C containing 5% CO_2_. Other cells were maintained in RPMI-1640 medium (Corning, Manassas, VA, USA) supplemented with 10% FBS (Corning) and 1X penicillin–streptomycin (Invitrogen, Carlsbad, CA, USA) at 37 °C containing 5% CO_2_.

### 4.3. Antibodies for Immunoblot and Immunocytochemistry

The following antibodies were used to detect target proteins in this study: PKCί (Cat. No. 2998), NOTCH3 (Cat. No. 5276), PARP (Cat. No. 9542), MYC (Cat. No. 5605), CCND1 (Cat. No. 2978), CCNA2 (Cat. No. 4656), and CCNB1 (Cat. No. 4138) from Cell Signaling Technology (Danvers, MA, USA), ELF3 (Cat. No. ab133621) from Abcam (Toronto, ON, Canada), and FOXM1 (Cat. No. sc-271746), CTNNB1 (Cat. No. sc-7199), p53 (Cat. No. sc-126), GAPDH (Cat. No. sc-25778), β-actin (Cat. No. sc-47778), and α-tubulin (Cat. No. sc-8035) from Santa Cruz Biotechnology (Dallas, TX, USA). Antibodies were applied to immunoblots at 1:1000 dilutions in 5% skim milks/1x Tris-buffered saline (TBS). 

### 4.4. ELF3 and FOXM1 Expression Knockdown

ELF3 expression was silenced by transfecting with ELF3-specific siELF3 (Cat. No. SI04265660, Qiagen, Hilden, Germany) targeting the sequence 5′-CTGGACTGGATCAGCTACCAA-3′, FOXM1-specific siFOXM1 (Cat. No. SI04140808, Qiagen, Hilden, Germany) targeting the sequence 5’-AACATCAGAGGAGGAACCTAA-3′, (Supplementary Table S4) and Allstar NC siRNA (Cat. no. SI03650318, Qiagen, Germany) at 5 nM using Lipofectamine RNAiMAX (Cat. no. 13778150, Thermo Fisher Scientific, San Jose, CA, USA). siRNA transfections were done according to manufacturer’s instructions. For immunoblots, A549 and H1975 (2.0 × 10^5^) were seeded in 60 mm dishes, and siRNA-treated cells were harvested at 3 time points (24, 48, and 72 h) after siRNA transfection. For WST-1 assay, A549 and H1975 (3.0 × 10^3^) were seeded in a 96-well plate. Cells were incubated for 72 h before WST-1 assay (Cat. no. MK400, Takara, Japan). For measuring changes of cell viability, A549 and H1975 cells (3.0 × 10^3^) were seeded in a 96-well plate. Auranofin and gefitinib were treated for 48 h and OD was measured in 450 nm.

### 4.5. Treatment of Auranofin and Gefitinib

Auranofin (Cat. No. A6733) and gefitinib (Cat. No. SML1657) were purchased from Sigma-Aldrich (St. Louis, MO, USA). Auranofin and gefitinib were dissolved in dimethyl sulfoxide (DMSO) (Cat. no. 0231, Amresco, Solon, OH, USA). The following concentrations were used in in vitro treatment: auranofin 0.5, 1.0, 2.0, and 3.0 μM, gefitinib 5.0 and 10 μM. A549 and H1975 cells (2.0 × 10^6^) were seeded in 100 mm dishes. Cells were incubated for 48 h and harvested at the end time.

### 4.6. Quantitative Reverse Transcription PCR

Total RNA was extracted and purified with a RNeasy kit (Qiagen, Hilden, Germany) following the instructions in the manufacturer’s handbook. For synthesizing cDNA from total mRNA, a PrimeScript 1st Strand cDNA Synthesis kit (Takara Bio, Otsu, Japan) was used. Semi-quantitative PCR was performed using primers specific for the *ELF3* gene (forward: 5′-CCC AGC TCC TTT CTC CTG TG-3′, reverse: 5′-TGT GTC TGT AAG CCC ACA CC-3′), the *FOXM1* gene (forward: 5′-ATC TCA GCA CCA CTC CCT TG-3′, reverse: 5′-CTT GCT GAG GCT GTC ATTCA-3′), the *CCND1* gene (forward: 5’-TGA GGG ACG CTT TGT CTG TC-3’, reverse:5’-CTT CTG CTG GAA ACA TGC CG-3’), and the 18S rRNA (forward: 5’-GTA ACC CGT TGA ACC CCA TT-3′, reverse: 5’-CCA TCC AAT CGG TAG TAG CG-3′). The 18S rRNA was used as an internal control gene for several target genes (Supplementary Table S5). Amplification was carried out (*n* = 3) on a LC480 real-time PCR system (Roche, Basel, Switzerland) as described in a previous report [33].

### 4.7. Analyses of Cell Cycle and Apoptosis

A549 and H1975 cells (1.0 × 10^5^) were seeded in 6-well plates. For cell cycle assay, cells were trypsinized, collected by centrifugation, and resuspended in PBS. Cells were fixed in 70% ethanol and incubated at 4 °C for overnight. The cells were centrifuged and incubated with FxCycleTMPI/RNase Staining Solution (Cat. No. F10797, Invitrogen, Carlsbad, CA, USA) for 30 min. Cell cycle assay was analyzed by FACS-LSR Fortessa (BD, San Jose, CA, USA). 

A549 and H1975 cells (8.0 × 10^4^) were seeded in 6-well plates. Apoptosis assay was analyzed by FACS-Verse using BD FITC Annexin-V Apoptosis Detection Kit I (Cat No.556547, BD Biosciences, San Diego, CA, USA) according to the manufacturer’s proto-col.

### 4.8. In Vivo Analysis of Auranofin-Gefitinib Combination

A549 and H1975 (5.0 × 10^6^) cells were subcutaneously injected in 5-week-old male BALB/C nude mice (CAnN.Cg-Foxn1nu/CrljOri, Orient Bio, Seongnam, Korea). When the tumor volume reached over 300 mm^3^ (tumor volume = [width^2^ × length]/2) for 3–4 weeks, auranofin and gefitinib were injected intraperitoneally. The following concentrations of auranofin and gefitinib: auranofin 2.5 mg/kg (3.7 μM equivalently) and 5.0 mg/kg (7.4 μM equivalently) [34], gefitinib 10 mg/kg (22 μM equivalently) [35]. Stock solution of auranofin and gefitinib were dissolved in dimethyl sulfoxide (DMSO). Before intraperitoneal injection into mice, the final concentration was adjusted within 50 μL of injection volume. Auranofin and gefitinib were injected 4 times per 48 h for 8 days. Mice were sacrificed on the following day of the last injection. This study was approved by the Institutional Animal Care and Use Committee (IACUC) of National Cancer Center (NCC-21-603).

### 4.9. Statistical Analysis

The statistical significance of differences between groups was determined using an χ^2^-test and Student’s *t*-test. *p*-values < 0.05 were considered statistically significant.

## 5. Conclusions

Our results provide an alternative therapeutic strategy to overcome TKI resistance in patients harboring clinically important mutations in EGFR or K-RAS. As described above, the inactivation of ELF3 increased the rate of cell death and decreased FOXM1 levels in NSCLC with K-Ras and EGFR T790M/L858R mutations in vitro and in vivo. Our results suggest that ANF may potentially be used as an effective alternative drug to overcome EGFR TKI resistance.

## Data Availability

Not applicable.

## Supplementary Materials

The following are available online at https://www.mdpi.com/article/10.3390/ijms222212287/s1.
