# Supplementary material for: ELF3 Is a Target That Promotes Therapeutic Efficiency in EGFR Tyrosine Kinase Inhibitor-Resistant Non-Small Cell Lung Cancer Cells via Inhibiting PKCί
Source: Int J Mol Sci. 2021 Nov 13;22(22):12287. doi: 10.3390/ijms222212287 (PMC8620479; doi:10.3390/ijms222212287)
Supplement: Supplementary file 1 [file ijms-22-12287-s001.zip › ijms-1446678-supplementary.pdf]

Supplementary Information

ELF3 is a target that promotes therapeutic efficiency in EGFR tyrosine kinase inhibitor-resistant non-small cell lung cancer cells via inhibiting PKCí

Jeon-Soo Lee [1], Young Eun Choi [1], Sunshin Kim[2], Ji-Yeon Han [2], and Sung-Ho Goh [1,*]

[1] Division of Cancer Biology, Research Institute, National Cancer Center, Korea; raphael@ncc.re.kr (J.S.L.); 75162@ncc.re.kr (Y.E.C.); andrea@ncc.re.kr (S.H.G.)
[2] Division of Rare and Refractory Cancer, Research Institute, National Cancer Center, Korea; ksunshin@ncc.re.kr (S.K.); jymama@ncc.re.kr (J.Y.H.)
* Correspondence: andrea@ncc.re.kr; Tel.: (S.H.G.)

Supplementary Table S1. Clinical information of patient derived cells.

DC	Sex	Age	Histology[1]	Driver mutation	Source[2]	Disease stage[3]
NCCLu-009	M	60	NSCLC-SaC	-	PE	None
NCCLu-025	M	53	NSCLC-ADC	-	PCE	PD
NCCLu-041	F	41	NSCLC-ADC	EGFR L858R	PE	PD
NCCLu-045	F	62	NSCLC-ADC	EGFR L858R	PE	PD
NCCLu-049	F	62	NSCLC-ADC	EGFR L858R	PE	PD
NCCLu-064	M	71	SCLC-ED	-	PE	PR
NCCLu-088	M	71	SCLC-ED	-	PE	PD
NCCLu-089	F	37	NSCLC-ADC	ALK fusion	PE	SD
NCCLu-096	M	51	NSCLC-ADC	EGFR ex19del	PE	PD
NCCLu-107	F	50	NSCLC-ADC	EGFR ex19del	ASC	PD
NCCLu-119	F	37	NSCLC-ADC	ALK fusion	PE	PD
NCCLu-128	F	77	NSCLC-ADC	EGFR L858R	PE	PR

[1] ADC-adenocarcinoma, SaC-sarcomatoid, SCLC-ED: small-cell lung cancer extensive stage disease; [2]PE-pleural effusion, PCE-pericardial effusion, ASC-ascites; [3]PD: progressive disease, SD-stable disease, PR-partial response

Supplementary Table S2. Sequences information of siRNAs

siRNA	Cat. No.[1]	Lot. No.	Target Sequence
siELF3	SI04265660	20111207	5′-CTGGACTGGATCAGCTACCAA-3′
siFOXM1	SI04140808	314574808	5'-AACATCAGAGGAGGAACCTAA-3′

Supplementary Table S3. Sequences information of RT-PCR primers

siRNA	Target Sequence
ELF3-Forward	5′-CCC AGC TCC TTT CTC CTG TG-3′
ELF3-Reverse	5′-TGT GTC TGT AAG CCC ACA CC-3′
FOX M1-Forward	5′-ATC TCA GCA CCA CTC CCT TG-3′
FOX M1-Reverse	5′-CTT GCT GAG GCT GTC ATT CA-3′
CCND1-Forward	5'-TGA GGG ACG CTT TGT CTG TC-3'
CCND1-Reverse	5'-CTT CTG CTG GAA ACA TGC CG-3'
ACTB-Forward	5′-CAT GTT TGA GAC CTT CAA CAC CCC-3′
ACTB-Reverse	5′-GCC ATC TCC TGC TCG AAG TCT AG-3′

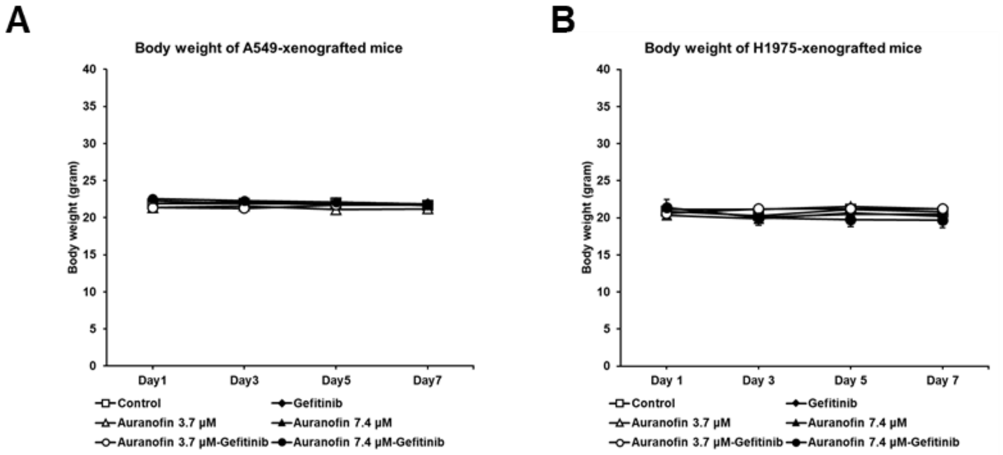

Supplementary Figure S1. Body weight changes of A549 (A) and H1975 (B) xenograft mice during the treatment of ANF, GEF, and ANF/GEF combination.
